# Caspase-Mediated Apoptosis in the Cochleae Contributes to the Early Onset of Hearing Loss in A/J Mice

**DOI:** 10.1177/1759091415573985

**Published:** 2015-03-26

**Authors:** Xu Han, Ruli Ge, Gang Xie, Ping Li, Xin Zhao, Lixiang Gao, Heng Zhang, Oumei Wang, Fei Huang, Fengchan Han

**Affiliations:** 1 Key Laboratory for Genetic Hearing Disorders in Shandong, Binzhou Medical University, Yantai, P. R. China; 2 Transformative Otology and Neuroscience Center, Binzhou Medical University, Yantai, P. R. China; 3 Department of Neurology, University Hospital of Binzhou Medical University, Binzhou, P. R. China

**Keywords:** ABR, DPOAE, age-related hearing loss, mouse model, hair cell, apoptosis

## Abstract

A/J and C57BL/6 J (B6) mice share a mutation in *Cdh23* (*ahl* allele) and are characterized by age-related hearing loss. However, hearing loss occurs much earlier in A/J mice at about four weeks of age. Recent study has revealed that a mutation in citrate synthase (*Cs*) is one of the main contributors, but the mechanism is largely unknown. In the present study, we showed that A/J mice displayed more severe degeneration of hair cells, spiral ganglion neurons, and stria vascularis in the cochleae compared with B6 mice. Moreover, messenger RNA accumulation levels of c*aspase-3* and *caspase-9* in the inner ears of A/J mice were significantly higher than those in B6 mice at 2 and 8 weeks of age. Immunohistochemistry localized caspase-3 expression mainly to the hair cells, spiral ganglion neurons, and stria vascularis in cochleae. *In vitro* transfection with *Cs* short hairpin RNA (shRNA) alone or cotransfection with *Cs* shRNA and *Cdh23* shRNA significantly increased the levels of caspase-3 in an inner ear cell line (HEI-OC1). Finally, a pan-caspase inhibitor Z-VAD-FMK could preserve the hearing of A/J mice by lowering about 15 decibels of the sound pressure level for the auditory-evoked brainstem response thresholds. In conclusion, our results suggest that caspase-mediated apoptosis in the cochleae, which may be related to a *Cs* mutation, contributes to the early onset of hearing loss in A/J mice.

## Introduction

Age-related hearing loss (AHL), or presbycusis, is the most common sensory deficit of the elderly population which adversely affects the quality of life for these individuals (Mulrow et al., 1990). Mouse models play a crucial role in understanding the pathogenesis associated with genetic hearing disorders (Angeli et al., 2012). The C57BL/6 J (B6) mouse strain is the most widely used mouse model for the study of aging and age-associated diseases. It is well known that hearing loss occurs at about nine to 12 months of age in B6 mice. Genome-wide linkage analyses identified an associated locus (named *ahl*) in the D10Mit5—D10Mit31 interval on Chromosome (Chr)10 and further genetic mapping delimited the interval to an 830 kilobase (kb) region on Chr10 (Zheng & Johnson, 2001; Noben-Trauth et al., 2003). Sequencing of genes in this interval identified a functional polymorphism (G753A) in the coding sequence of cadherin 23 (*Cdh23*). This single nucleotide polymorphism (SNP) occurs at the last position of exon 7 and alters the consensus splice site leading to in-frame skipping of exon 7. Inbred strain variants of the *Cdh23* gene have been shown to influence the onset and progression of AHL in mice (Kane et al., 2012).

A/J mice and B6 mice share the same *ahl* allele (Noben-Trauth et al., 2003). However, mice of the A/J substrain exhibit an early-onset progressive hearing loss with elevated auditory-evoked brainstem response (ABR) thresholds by 25 days of age which progresses to near deafness by 3 months of age (Zheng et al., 1999; Zheng et al., 2009). Therefore, additional genetic factors must be involved. Sequencing of the mitochondrial genome revealed a single nucleotide insertion in the tRNA-Arg gene (*mt-Tr*) that is likely responsible for the phenotypic effect (Johnson et al., 2001). However, the effect of the *ahl* locus combined with the mitochondrial effect is still not enough to account for the full extent of hearing loss exhibited by A/J mice. Linkage backcross was used to map yet another AHL locus (named *ahl4*) to the distal region of Chr10 (Zheng et al., 2009). As was the case with *mt-Tr,* the *ahl4* effect on hearing loss was limited to backcrossed mice with predisposing *ahl/ahl* genotypes. The *ahl4* locus, which could explain about 40% of the ABR threshold variation in these mice, was recently identified as a mutation in the gene of *Cs* (Noben-Trauth & Johnson, 2009; Johnson et al., 2012).

In this study, the possible mechanism for gene mutations that contribute to earlier onset of hearing loss in A/J mice was explored in the following aspects: (a) phenotypes of A/J mice found by a time course observation; (b) transcription levels of apoptotic-related genes in the inner ears of A/J mice; (c) caspase-3 expression in an inner ear cell line (HEI-OC1) by knockdown of only the *Cs* gene or in combination with the *Cdh23* gene; and finally, (d) improvement of hearing in A/J mice using a pan-caspase inhibitor. The results indicated that caspase-mediated apoptosis in the cochleae contributes to early onset of hearing loss in A/J mice.

## Materials and Methods

### Mouse Preparation

Mice were originally purchased from the Model Animal Research Center of Nanjing University (Nanjing, P.R. China) and were relocated to Binzhou Medical University (Yantai, Shandong, P.R. China) for breeding in specific pathogen-free animal rooms. A total of 157 A/J mice (83 male and 74 female) and 137 B6 mice (75 male and 62 female) with ages from 2 to 46 weeks were used in this study. The animal studies were conducted in accordance with the principles set forth in the Guide for the Care and Use of Laboratory Animals of Binzhou Medical University and were approved by the Institutional Animal Use and Care Committee of Binzhou Medical University.

### Genotyping Mice for SNP of Cs gene

The unique A/J variant (c163a) is predicted to cause an amino acid change of histidine to asparagine at position 55 (H55N; Johnson et al., 2012). Based on the mutation, a genotyping method was developed by polymerase chain reaction (PCR; forward primer, 5′-GGAAACCCTATAGATTTTTCTTCCA-3′, reverse primer, 5′-AGAGCTCTTACCAAGTCCACAG-3′), followed by *Nla III* restriction endonuclease digestion of the PCR products. Genomic DNA was prepared from mouse tail tips, and the PCR reaction was performed in a 20 μl reaction mixture, as described previously. Five microliters of the PCR products were subjected to *Nla III* restriction endonuclease (New England BioLabs) digestion and agarose gel electrophoresis.

### Measurement of ABR Thresholds

ABR was measured at various intervals at 3, 4, 10, 12, 28, and 42 weeks for B6 and A/J mouse strains. A computer-aided evoked potential system (Intelligent Hearing Systems, Miami, FL, USA) was used to test mice for ABR thresholds, as described previously (Johnson et al., 1997; Zheng et al., 1999). The amplified brainstem responses were averaged by a computer and displayed on a computer screen. Auditory thresholds were obtained for each stimulus (namely clicks and 8 -, 16 -, and 32-kHz tone-bursts) by reducing the sound pressure level at 10-decibel steps and finally at 5-decibel steps up and down to identify the lowest level at which an ABR pattern could be recognized.

### Test of Distortion Product Oto-acoustic Emission

To test the function of outer hair cells (OHC) in different mice at different time points, we used the IHSS Smart EP 3.30 and USB ez Software (Intelligent Hearing Systems) for distortion product oto-acoustic emission (DPOAE) measurement, which was conducted for pure tones from 4 to 35 kHz (Polak et al., 2004; Han et al., 2012). Frequencies were acquired with an F2:F1 ratio of 1.22. The stimuli were presented starting from the lowest frequencies and increasing to the highest frequencies tested.

### Observation of OHC in the Cochleae

The organ of Corti was carefully microdissected out and subdivided into base turn, middle turn, apex turn, and mounted in Shandon immu-mount (Thermo) on glass slides. The surface preparations were first stained for F-actin with Alexa Fluor 488 conjugated to phalloidin (1:500 dilution) for 1 hr at room temperature, protected from light, and then mounted after washing thrice in 1 × phosphate-buffered saline (PBS), and lastly observed with a confocal fluorescence microscope (Leica DMI4000 B, Leica Microsystems, Wetzlar, Germany). Hair cells were counted as present if V-shapes of hair bundles were intact. OHC and inner hair cell (IHC) counts were made in the three turns of the organs of Corti in A/J and B6 mice and were displayed as percentage of hair cell missing.

### Histological Analyses of Inner Ears

Histological analyses of inner ears were performed following the methods described previously (Zheng et al., 2009). The time points selected were 2 weeks, 4 weeks, 10 weeks, and 46 weeks. Sections (5 µm) mounted on glass slides were counterstained in hematoxylin and eosin (HE). Hair cells, spiral ganglion cells, and stria vascularis were observed under light microscopy (Leica DMI4000 B, Leica Microsystems, Wetzlar, Germany). Spiral ganglion cell counts were carried out following the methods described previously (Guillery, 2002; Semaan et al., 2013). The average value in four sections from a turn of each animal’s cochlea was defined as the density for that turn. The width of stria vascularis was measured with the aid of ImageJ software. The mean value in four sections from a turn of each animal’s cochlea was defined as the width for this stria vascularis.

### Assays for Gene Transcription

A/J mice (*n* = 4–6) and B6 littermates (*n* = 4–6) at the ages of 2 and 8 weeks were randomly chosen. Both bullae from each mouse were removed. Total RNA was extracted using Trizol (Invitrogen, Carlsbad, CA, USA), and cDNA was made using random primers following the First-Strand Synthesis protocol (Invitrogen). Real-time PCR was used to determine relative gene expression levels using FastStart Universal SYBR Green Master (Roche Diagnostics, Indianapolis, IN, USA) and MyiQ™ Real-Time PCR Detection System (Bio-Rad Laboratories, Inc., Hercules, CA). The messenger RNA (mRNA) level of glyceraldehyde 3-phosphate dehydrogenase (*Gapdh*) was used as the endogenous control. Primers used-*Gapdh:* forward, 5′-CTTCCGTGTTCCTACCCCCAATGT-3′, reverse*,* 5′-GCCTGCTTCACCACCTTCTTGATG-3′; *Cs*: forward, 5′-CCAACCAATCTGCACCCTAT-3′, reverse, 5′-AATGAGGTCCATGCAGTCCT-3′; *Cdh23*: forward, 5′-TGACACGTACCTGCTCATCA-3′, reverse, 5′-CCTTGGTGGTCACTGACAGA-3′. *caspase-3*: forward, 5′-TGTCATCTCGCTCTGGTACG-3′, reverse, 5′-AAATGACCCCTTCATCACCA-3′; *caspase-9*: forward, 5′-CCTAGTGAGCGAGCTGCAAG-3′, reverse, 5′-ACCGCTTTGCAAGAGTGAAG-3′; apoptosis-inducing factor (*Aif*): forward, 5′-CTGCTCAGGACCTGCCTAAT-3, reverse, and 5′-CCGTTGCAATCAAGCACTTT-3′. All primers were synthesized by Sangon Biotech Co. Ltd. (Shanghai, China). Each PCR reaction contained 12.5 μl of FastStart Universal SYBR Green Master Mix, 1 μl of cDNA, 2 μl of each 5 μM primer and 7.5 μl of ddH_2_O. The reaction conditions were as follows: denature at 95℃ for 3 min, followed by 40 cycles of 94℃ for 15 s, 60℃ for 30 s, and 72℃ for 40 s, and a final extension at 72℃ for 10 min. The fold-change in genes relative to *Gapdh* was determined by the 2^(-ΔΔCt)^ method (Semaan et al., 2013).

### Immunostaining for Active Caspase-3

A time course immunocytochemistry study of caspase-3 expression was carried out for A/J and B6 mice (four for each group at each time point). The time points selected were 2, 4, 10, and 46 weeks. Paraffin sections (5 µm) were made, as described previously (Han et al., 2012), except that the inner ears were fixed in 4% paraformaldehyde and decalcified with 10% EDTA. After dewaxing, the sections were subjected to heat-induced antigen retrieval in citrate buffer (0.01 mol/L, PH 6.0), washed in 1 × PBS at room temperature thrice for 5 min, and permeabilized in 0.5% Triton X-100 for 15 min. After being washed thrice in 1 × PBS for 5 min and blocked in 3% goat serum and 2% bovine serum albumin at 37℃ for 1 hr, the samples were immersed in rabbit anticleaved caspase-3 (Asp175) antibody (1:400 dilution) (Cell Signaling Technology, Inc., Danvers, MA, USA) and incubated at 4℃ overnight. Samples with primary antibody omitted were used as the negative controls. After being washed thrice in 1 × PBS for 5 min, the samples were immersed in anti-rabbit secondary antibody- Alexa 488 (1:800 dilution) at 37℃ for 1 hr, and then counterstained with Hoechst33342 (10 µg/ml in PBS) for 15 min at room temperature. Finally, the sample mounts were observed under immunofluorescent confocal microscopy (Leica DMI4000 B, Leica Microsystems, Wetzlar, Germany).

### Caspase-3 Expression in HEI-OC1 Cells Transfected With shRNA

Transfection of short hairpin RNA (shRNA) was carried out following the protocol provided (Santa Cruz Biotechnology). Five groups were prepared, each with 0.4 × 10^6^ cells in triplicate: control group (2 µg of shRNA plasmid A was applied), copGFP group (2 µg of GFP (green fluorescent protein) control plasmid), *Cs* knockdown group (2 µg *of Cs* shRNA plasmid), *Cdh23* knockdown group (2 µg of *Cdh23* shRNA plasmid), and *cdh23 + Cs* knockdown group (1 µg of *Cdh23* shRNA plasmid and 1 µg of *Cs* shRNA plasmid). Briefly, HEI-OC1 cells were cultured at 33℃ in Dulbecco’s modified Eagle medium (DMEM/High glucose, Hyclone) supplemented with 10% fetal bovine serum (FBS; 16000-044, Gibco) in a humidified incubator with 10% CO_2_. When the cells reached 60% confluency in the culture dishes, the cells were transfected with *Cs* shRNA (*Cs* shRNA Plasmid, sc-96228-SH, Santa Cruz Biotechnology), *Cdh23* shRNA (cadherin 23 shRNA Plasmid, sc-43009-SH, Santa Cruz Biotechnology) or *Cs* shRNA and *Cdh23* shRNA to knockdown the transcription levels of *Cs* and *Cdh23* genes. 48 hr later, the transfection efficiency of copGFP group was examined under florescence microscopy. Transcription levels of *Cdh23* and *Cs* in the four groups were evaluated by real-time reverse transcription (RT)-PCR (as described above). Meanwhile, Western blotting was carried out to detect the expression levels of caspase-3. Cells from each group were washed with ice-cold PBS (0.01 M pH 7.2–7.3) and lysed in 150 μl of ice-cold lysis buffer with protease inhibitors at 4℃ for 30 min. The mix was then centrifuged at 12000 rpm at 4℃ for 5 min. A BCA protein assay reagent kit (Beyotime, 20201ES76) was used to determine protein concentration in the supernatant. Western blotting was then performed, as described previously (Park et al., 2012). Extracted protein (40 µg/lane) of each group was separated by 15% sodium dodecyl sulfate-polyacrylamide gel electrophoresis and then transferred electrophoretically onto a polyvinylidene difluoride (PVDF) membrane (Millipore). After blocking with 5% nonfat milk at room temperature for 1.5 hr, the PVDF membrane was incubated with the rabbit anti-mouse primary antibodies against caspase-3 (ab2302,1:200 dilution, Abcam) at 4℃ overnight. After washing three times in TBST (a mixture of tris-buffered saline and Tween 20), the PVDF membrane was incubated with the horseradish peroxidase-conjugated goat anti-rabbit secondary antibody (ab97051, 1:5000 dilution, Abcam) at room temperature for 1 hr. After rinsing in TBST, the proteins were detected using LumiPico(R) ECL Kit (ShineGene, Shanghai) and photographed in a chemiluminescence instrument (Clinx science, 3100Mini, 90175). Meanwhile, β-actin was detected with rabbit anti-mouse primary antibody (1:2000 dilution, Immunoway). The gray level of each band was quantified using the ImageJ software. The relative protein levels of active caspase-3 were calculated when corrected with those of the corresponding β-actin. The experiments were performed in triplicate.

### Otoprotection by Antiapoptosis in A/J Mice

Twenty-one A/J mice at the age of 7 days were divided into three groups: a test group (six mice), a dimethyl sulfoxide (DMSO) group (six mice), and an untreated group (nine mice). In the test group, the mice were injected intraperitoneally (IP) under sterile conditions with Z-VAD-FMK (1 µg/µl) (Z-Val-Ala-Asp(OMe)-Fluoromethylketone (Catalog No.1140-5), BioVision Incorporated, Milpitas, CA, USA) in DMSO at the dosage of 1.5 µg/g mouse weight: first, 10 injections, once every other day; second, five injections, once every 3 days; and lastly, four injections every 4 days until the time of euthanization for experimental purposes at the age of 8 weeks. Mice in the DMSO group received 1.5 µl of DMSO per gram of mouse weight at the same time points as the test group. ABR and DPOAE were tested at 3, 4, 6, and 8 weeks for the mice in each group, and then all of the mice from each group were sacrificed for histological investigation.

### Statistical Analysis

Analysis of variance test was used for analysing data of ABR thresholds, DPOAE amplitudes, gene transcription levels, spiral ganglion cell counts, and widths of stria vascularis. Data of hair cell loss were analyzed by χ^2^ test; *p* < .05 was considered to be significant.

## Results

### Characterization of the Hearing Loss in A/J Mice by a Time Course Observation

**Figure 1.**
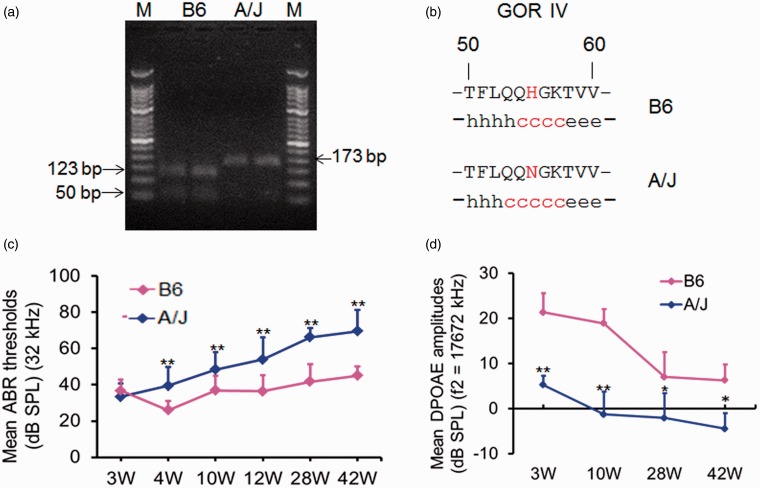
Characterization of hearing loss in A/J mice by a time course observation. (a) 2.5% agarose gel electrophoresis of the PCR products digested with *Nla III* restriction endonuclease. Lane M, 50 bp DNA ladders; two lanes designated as B6: The *Nla III* restriction endonuclease cutting the 173 bp PCR fragment from *Cs* gene in B6 mice into two distinct fragments (123 bp and 50 bp); two lanes designated as A/J: *Cs* mutation in *A/J* mice alters the restriction site and leaving only one band (173 bp). (b) The mutation changes the local secondary structure of *Cs* from an alpha helix (h) to a random coil(c) as predicted by GOR IV methods. (c) ABR threshold detection in A/J mice at ages of 3 weeks (*n* = 6), 4 weeks (*n* = 11), 10 weeks (*n* = 8), 12 weeks (*n* = 10), 28 weeks (*n* = 7), and 42 weeks (*n* = 7) and B6 mice with the corresponding age groups (*n* = 6, *n* = 10, *n* = 20, *n* = 13, *n* = 6, and *n* = 6, respectively). Each point represents the mean ABR threshold for a group, with error bar indicating *SD* from the mean. The results showed that ABR thresholds were significantly higher in the A/J mice than those of the B6 mice at stimulus frequencies of 32 kHz at all points of time. **p < .01. (d) DPOAE measurement in B6 (*n* = 6–10) and A/J (*n* = 6–10) mice with age from 3 to 42 weeks at f2 frequency of 17267 kHz. DPOAE amplitudes were significantly lower in A/J mice than those of B6 mice at 3, 10, 28, and 42 weeks of age, respectively. W: weeks; *p < .05; **p < .01. PCR = polymerase chain reaction; ABR = auditory-evoked brainstem response; DPOAE = distortion product oto-acoustic emission; SPL = sound pressure level.

The unique A/J variant (c163a) is predicted to cause an amino acid change of histidine to asparagine at position 55 (H55N). The mutation changes a *Nla III* restriction site from CATG as in B6 mice to AATG in A/J mice, leading to un-cutting of the PCR products from the genomic DNA of A/J mice. Therefore, a genotyping method was developed by PCR and *Nla III* restriction endonuclease digestion of the PCR products (Figure 1(a)). The mutation changed the local secondary structure of *Cs* from an alpha helix to a random coil as predicted by GOR IV (Garnier, Osguthorpe and Robson IV) methods (Figure 1(b)). A/J mice (male 22, female 17, *n* = 6–11 for each group) and B6 mice (male 29, female 19, *n* = 6–20 for each group) ranging in age from 3 to 42 weeks of age were assessed for ABR thresholds at stimulus frequencies of click, 8 kHz, 16 kHz, and 32 kHz. Overall, there was no significant difference for the ABR thresholds between A/J and B6 mice until 4 weeks of age. The levels of A/J mice were significantly higher (*p* < .05 or *p* < .01) than those of B6 mice at different time points at different stimulus frequencies, though ABR thresholds of both mouse strains increased with age. Typical results at a stimulus frequency of 32 kHz are shown in Figure 1(c) and at click, 8 kHz, 16 kHz are shown in Figure S1. To evaluate the function of OHC, A/J and B6 mice at age ranging from 3 to 42 weeks were subjected to the DPOAE test. The amplitudes of DPOAE were significantly lower (*p* < .05) in A/J mice than those of B6 mice at f2 frequencies from 8844 Hz to 17672 Hz at each time point (*n* = 6–17). However, both strains showed a tendency of decrement in DPOAE amplitudes in this period (Figure S2). Typical DPOAE amplitudes in A/J mice and B6 mice at f2 frequency of 17672 Hz are shown in Figure 1(d).

### Histological Examination Reveals Early Onset, Progressive OHC Loss and Degeneration of Spiral Ganglion Neurons and Stria Vascularis in A/J Mice

**Figure 2.**
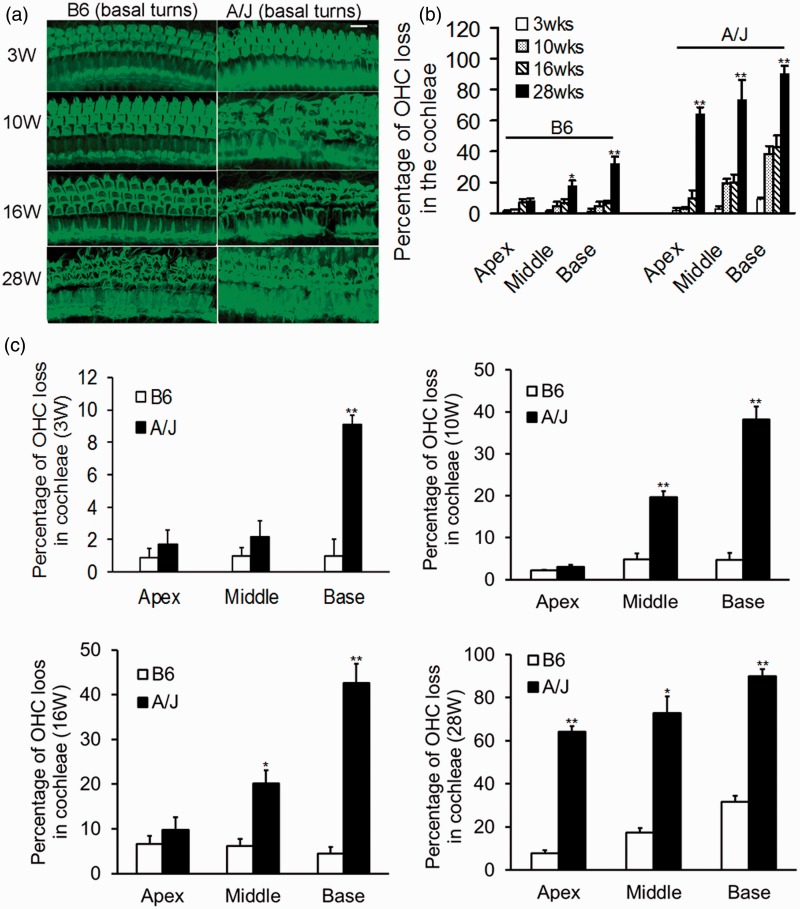
OHC loss in the cochleae of A/J mice. (a) Representative whole-mount alexa-fluor-488 phalloidin-stained preparations from basal turns of cochleae. OHC loss and abnormal morphology and arrangement of hair cells in A/J mice occur at 3 weeks of age (right), compared with the corresponding area in B6 mice (left). Both OHC and IHC loss was severe in A/J mice at age of 10 weeks or older. B6 mice also showed hair cell loss and abnormal arrangement, evidently at 28 weeks of age. Scale bars = 50 µm. (b) Progressive OHC loss in the tree turns of B6 and A/J mice at 3, 10, 16, and 28 weeks of age. OHC loss in each turn of both mouse strains increased with age. The percentages of OHC loss at 28 weeks of age were significantly more than any other time points, except for those at the apex turns in B6 mice. *p < .05; **p < .01. (c) A time course comparison of OHC loss between B6 and A/J mice. A/J mice have significantly more OHC loss beginning at 3 weeks of age at basal turns and becoming more severe at 28 weeks of age at the three turns, compared with those of B6 mice. (*n* = 3–5 for each group at each time point). *p < .05; **p < .01. W: weeks. OHC = outer hair cells; IHC = inner hair cell.

Lesion in the hair cell was presented as hair cell loss and abnormal cell morphology. B6 mice showed normal hair cell morphology and regular arrangement with stereocilia bundles exhibiting normal V-shaped morphology at 3, 10, and 16 weeks of age, and hair cell loss and abnormal arrangement at 28 weeks of age at the basal turns (Figure 2(a)). However, OHC loss and abnormal appearance of IHC morphology and arrangement in A/J mice were already evident at basal turns at 3 weeks of age, compared with those of B6 mice. A time course observation of OHC loss at apex, middle, and basal turns of cochleae in B6 and A/J mice are shown in Figure 2(b) and (c). Overall, OHC loss spread from base to the apex and was more severe in A/J mice than in B6 mice from 3 to 28 weeks of age.

**Figure 3.**
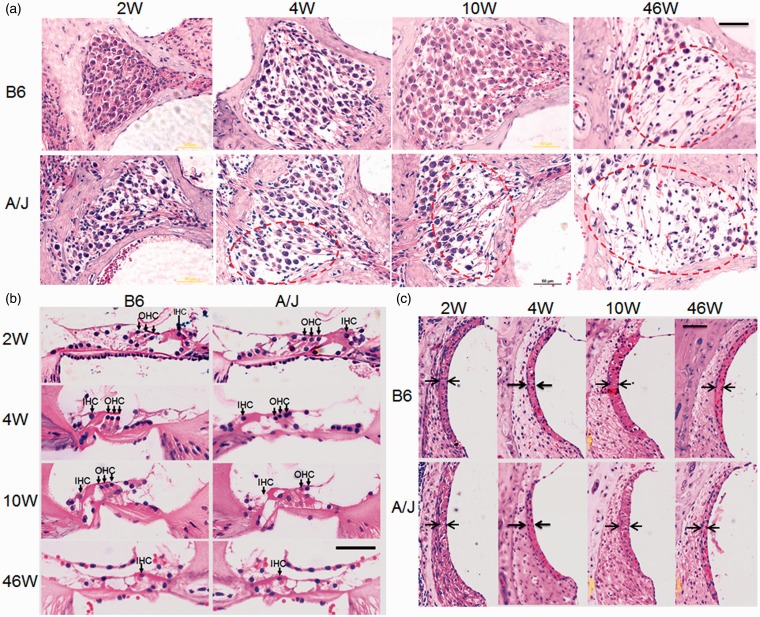
Typical pathology on HE sections from basal turns in the cochleae of A/J mice. (a) Pathological alteration in the spiral ganglion neurons (SGNs). B6 mice showed normal density of SGNs until 46 weeks of age. However, SGNs in the A/J mice were already sparse at 4 weeks of age, and loss of SGNs became severe at 46 weeks of age (indicated by circles). (b) Hair cell pathology in HE staining sections. OHC was intact before 46 weeks of age for B6 mice, whereas hair cell lesion became severe at 10 weeks of age for A/J mice (indicated by arrows). (c) Alteration of stria vascularis in A/J mice. The width of stria vascularis (indicated by arrows) of A/J mice was generally thinner than that of B6 mice at all the points of time, though there is significant difference only at 2 weeks of age (*p < *.05). W: weeks; scale bar = 50 µm for all panels. HE = hematoxylin and eosin; OHC = outer hair cells.

Meanwhile, pathological observation at age 2 to 46 weeks showed that A/J mice suffered from progressive loss of spiral ganglion neurons (SGNs) at the basal turns (Figure 3(a)). The average cell densities (cells/mm^2^) of the SGNs at the basal turns of A/J mice were lower than those of B6 mice at each time point. There was a significant difference between A/J and B6 mice at the age of 4 weeks (739 ± 76 vs. 1124 ± 345, *p* = .032) or 10 weeks (607 ± 161 vs. 838 ± 164, *p* = .048) and no significant difference (*p* > .05) at the age of 2 weeks (979 ± 367 vs. 1268 ± 331) or 46 weeks (561 ± 228 vs. 573 ± 231). A time course observation on HE staining sections also showed earlier progressive hair cell loss in A/J mice (Figure 3(b)). Moreover, A/J mice showed relative thinner stria vascularis from 2 to 46 weeks of age (Figure 3(c)). The mean width (µm) of the stria vascularis at the basal turns of A/J mice is 16.2 ± 2, 18.3 ± 2.5, 21.5 ± 1.1, and 13.8 ± 2, respectively, at 2, 4, 10, and 46 weeks of age, whereas that of B6 mice is 20 ± 2, 21.6 ± 2.8, 24.5 ± 1.5, and 16.6 ± 2.2, respectively, at the corresponding time point. There was significant difference between the two strains only at 2 weeks of age (*p* < .05). There was no statistical difference for the densities of SGNs or width of stria vascularis at the middle turns at any time point between the two mouse strains (data not shown).

### Upregulation of Apoptosis Related Genes in the Cochleae of A/J Mice

**Figure 4.**
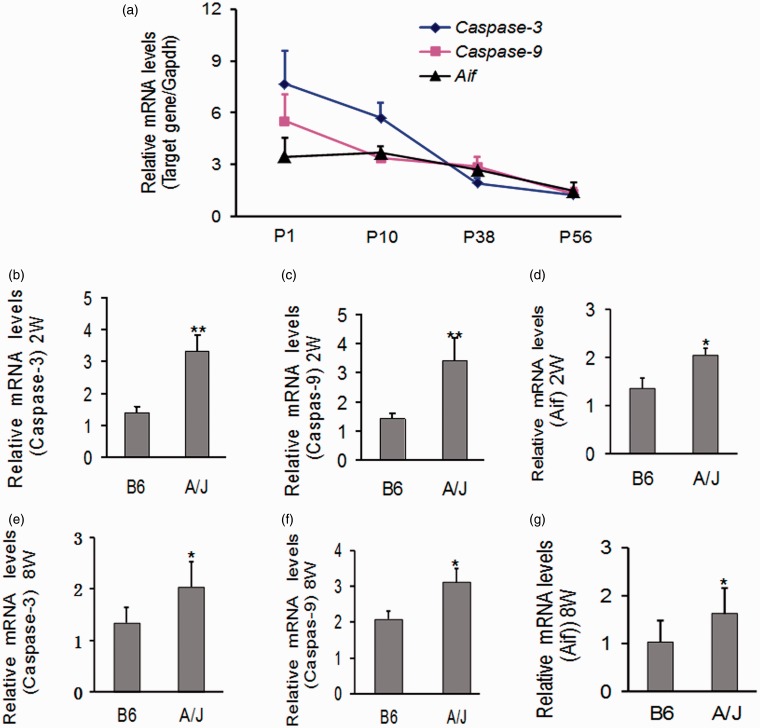
Relative mRNA levels of apoptosis related genes in the cochleae of A/J mice. (a) Time course mRNA accumulation level of caspase-3, caspase-9, and Aif, corrected by *Gapdh*, in the inner ears from A/J mice (*n* = 4–5 at each time point). mRNA levels of caspase-3 and caspase-9 at P1 were more than two times the levels at P56 but declined with time in this period. (b to g) Comparison of gene transcription levels between A/J mice and B6 mice. mRNA levels of caspase-3, caspase-9, and Aif in the inner ears from A/J mice (*n* = 4–6) were significantly higher than those from B6 mice (*n* = 4–6) at 2 weeks (b–d) or 8 weeks (e–g). Error bars represent the *SD* from the mean. W: weeks; *p < .05; **p < .01. mRNA = messenger RNA; *Gapdh = *glyceraldehyde 3-phosphate dehydrogenase.

Time course mRNA accumulation levels of *caspase-3*, *caspase-9*, and *Aif* in the inner ears from A/J mice (*n* = 4–5) were detected by real-time RT-PCR. Higher mRNA levels of *caspase-3* and *caspase-9* occurred at postnatal Day 1 (P1), indicating that signals of caspase-mediated apoptosis are strong in the inner ears of A/J mice even at birth. mRNA levels *of caspase-3, caspase-9*, and *Aif* were also detected in the inner ears of A/J and B6 mice (*n* = 4–6) at age of 2 weeks or 8 weeks. Transcription levels of the three genes from A/J mice were significantly higher than those from B6 mice at each time point (*p* < .01 or *p* < .05) (Figure 4). *In situ* observation for active caspase-3 expression in the cochleae of A/J and B6 mice showed that caspase-3 was mainly localized to hair cells, SGNs, and strial vascularis of both mouse strains. We failed to notice a significant difference in the staining intensity of caspase-3 in the cochleae between A/J mice and B6 mice at any time points (Figure S3).

### Cotransfection With Cs shRNA and Cdh23 shRNA Increase the Levels of Caspase-3 in HEI-OC1 Cells

**Figure 5.**
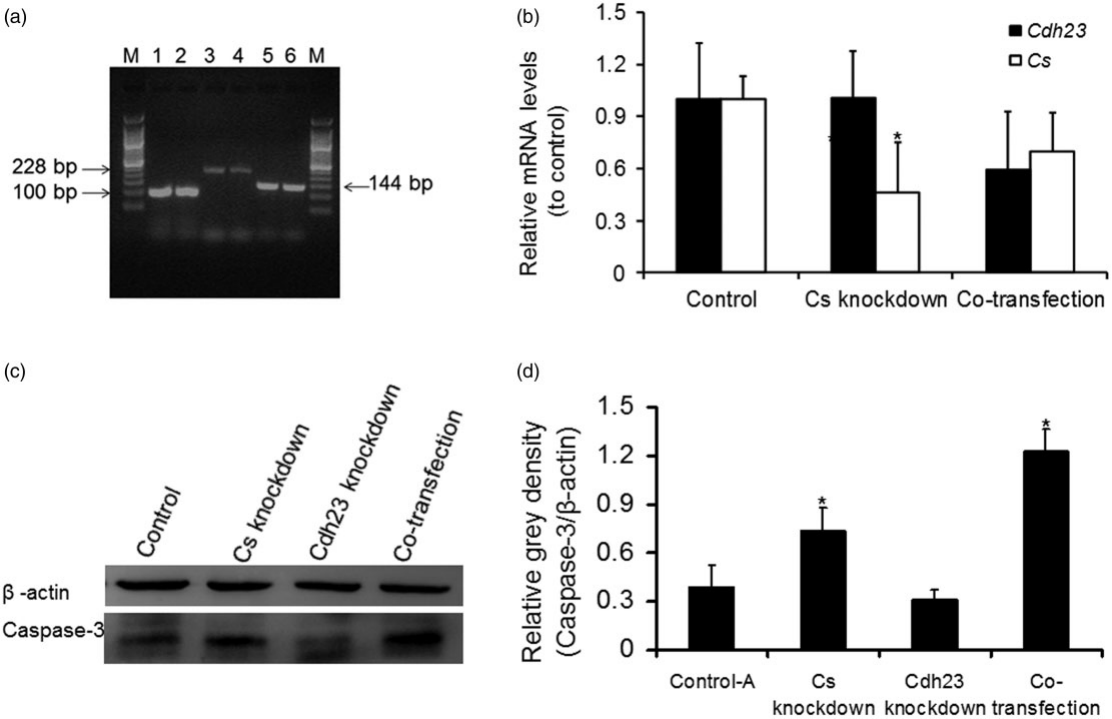
Expression of caspase-3 in HEI-OC1 cells. (a) 2% agarose gel electrophoresis of the RT-PCR products of *Gapdh, Cs*, and *Cdh23* in HEI-OC1 cells. Lane M, 50 bp DNA ladders; lanes 1–2, 3–4, and 5–6 represent RT-PCR products of *Gapdh* (100 bp), *Cdh23* (228 bp), and *Cs* (144 bp), respectively. (b) Transcription levels of *Cs* and *Cdh23* in HEI-OC1 cells transfected with shRNA. mRNA levels of *Cs* was significantly suppressed (*p* < .05) by *Cs* shRNA (*Cs* knockdown group) compared with that by control shRNA (control, limited to1 unit for the level of each gene). mRNA levels of *Cs* and *Cdh23* in cotransfencted group (*Cdh23* shRNA + *Cs* shRNA) were also lower than those of the controls. (c) Caspase-3 levels determined by Western blotting. Caspase-3 expression levels were markedly elevated in *Cs* knockdown group or cotransfected group compared with those of controls, whereas were not elevated in *Cdh23* knockdown group. (d) The average gray intensity of caspase-3 protein detected by Western blotting was standardized to that of β-actin. Caspase-3 expression levels in *Cs* knockdown group or cotransfected group were significantly higher than those of the controls. Data are shown as mean ± *SD* of triplicate experiments. *p < .05. shRNA = short hairpin RNA; RT-PCR = reverse transcription polymerase chain reaction; mRNA = messenger RNA; *Gapdh = *glyceraldehyde 3-phosphate dehydrogenase.

To demonstrate that a reduction in *Cs* and CDH23 expression leads to cell apoptosis, downregulation of the transcription levels of *Cdh23* and *Cs* genes by shRNA transfection was performed in HEI-OC1 cells. The results showed that the knockdown of *Cs* alone or *Cs* and *Cdh23* together reduced the transcription level of the corresponding gene and increased the levels of caspase-3 expression in the cells. Knockdown of *Cdh23* alone failed to increase caspase-3 in the cells (Figure 5).

### Otoprotection in A/J Mice by Z-VAD-FMK

**Figure 6.**
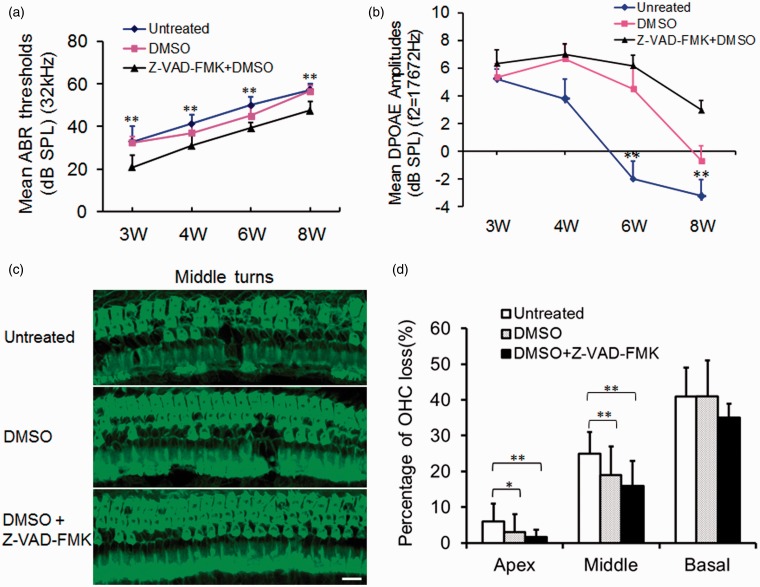
Otoprotection by pan-caspase inhibitor in A/J mice. (a) ABR thresholds in the three mouse groups measured at the stimulus frequency of 32 kHz. The number of mice tested was nine, six, and six for no treatment, DMSO and Z-VAD-FMK + DMSO groups, respectively. Each point represents the mean ABR threshold for a group, with error bar indicating *SD* from the mean. The results show that ABR thresholds were significantly lower in the Z-VAD-FMK + DMSO-treated mice than those of the untreated mice at the four time points (*p* < .01). There were significant differences for ABR thresholds between Z-VAD-FMK + DMSO-treated and DMSO-treated groups at all time points except at 4 weeks of age (*p* < .05); or between DMSO-treated and untreated mice at 6 weeks of age (*p* < .05). (b) A time course observation of DPOAE to stimulus frequency of 17672 kHz (f2) in the three mouse groups. Though gradually declined with time, the mean DPOAE amplitudes were significantly higher in Z-VAD-FMK + DMSO-treated mice than those in the untreated mice at age of 6 weeks or 8 weeks (*p* < .01). (c) Typical OHC distribution in the middle turns of cochleae in the three mouse groups. Loss of OHC was evident in the middle turns of cochleae of the untreated or DMSO-treated mice at 8 weeks of age, whereas only some OHC loss was seen in the corresponding areas of the Z-VAD-FMK + DMSO-treated mice. (d) Percentage of OHC loss in the three turns of cochleae in the three mouse groups at age of 8 weeks. W: weeks; Scale bar = 50 µm; *p < .05; **p < .01. ABR = auditory-evoked brainstem response; SPL = sound pressure level; DMSO = dimethyl sulfoxide; OHC = outer hair cells.

Z-VAD-FMK is a powerful, irreversible, and cell-permeable pan-caspase inhibitor that shows otoprotective or neuroprotective effects and suppresses the activity of caspases including caspase-3 (Mizutari et al., 2008; Han et al., 2012; Li et al., 2014). ABR thresholds in the three mouse groups were tested at 3, 4, 6, and 8 weeks of age. The results of ABR thresholds at a stimulus frequency of 32 kHz are shown in Figure 6(a) and at click, 8, 16 kHz are shown in Figure S4. Generally, Z-VAD-FMK + DMSO-treated mice displayed lower ABR thresholds compared with those of the DMSO-treated or untreated mice at all stimulus frequencies. ABR thresholds were significantly lower in Z-VAD-FMK + DMSO-treated mice than those of the untreated mice at stimulus frequencies of click, 8, 16, and 32 kHz at the four time points except for click at 3 weeks of age (*p* < .05 or *p* < .01). ABR thresholds in Z-VAD-FMK + DMSO-treated group were also significantly lower (*p* < .05) than those of the DMSO-treated group at stimulus frequencies of click at 6 and 8 weeks of age, at 8 kHz at the four time points, at 16 kHz at 8 weeks of age, or at 32 kHz for all time points except at age of 4 weeks. The ABR thresholds in the DMSO-treated mice were also significant lower than the thresholds of the untreated mice at 4 weeks of age at the stimulus frequencies of click and 16 kHz (*p* < .05) or at 6 weeks of age for all stimulus frequencies *(p* < .05), indicating that DMSO alone also provided hearing protection (Melki et al., 2010; Han et al., 2012).

DPOAE amplitudes were tested for the three mouse groups at 3, 4, 6, and 8 weeks of age (Figure S5). Representative results at f2 frequencies of 17672 Hz are shown in Figure 6(b). The mean amplitudes were significantly higher in Z-VAD-FMK + DMSO-treated mice than those in the untreated (*p* < .01) group at 6 and 8 weeks of age and was significantly higher than that in the DMSO-treated group at the age of 8 weeks (*p* < .01).There were also significant differences for the DPOAE amplitudes between the untreated group and DMSO-treated group at 6 weeks of age (*p* < .01), indicating DMSO also preserves OHC. However, DPOAE amplitudes gradually decreased with time in the untreated group, the DMSO-treated group, and Z-VAD-FMK + DMSO-treated group.

Loss of OHC was evident in the middle turns of cochleae of the untreated or DMSO-treated mouse groups at 8 weeks of age, whereas only some OHC loss was seen in the corresponding areas of the Z-VAD-FMK + DMSO-treated mice (Figure 6(c)). OHC loss was counted and averaged from apical to basal turn of the cochlear surface preparations in the three mouse groups (*n* = 4 per group) at 8 weeks of age. The mean percentages of OHC loss in the untreated group, DMSO-treated group, or Z-VAD-FMK + DMSO-treated group are shown in Figure 6(d). Percentage of OHC loss was significantly less in the Z-VAD-FMK + DMSO-treated group than that in the untreated group at the apical region or middle region of the cochlea (*p* < .05 or *p* < .01). There was also significantly less OHC loss in the DMSO-treated mice than that in the untreated mice at the middle turn (*p* < .05). IHC loss was not substantially altered by the treatment (data not shown). However, we failed to find significant differences among the percentages of OHC loss at the basal turns of cochleae in the three mouse groups. We considered that OHC loss at the basal turn of cochleae at 8 weeks of age was so severe that the effect of antiapoptotic treatment was limited.

## Discussion

A/J mice are used as a typical mouse model for AHL with mutiple gene mutations. In the present study, a time course observation from 3 to 42 weeks of age showed that ABR thresholds in the A/J mice were higher than the levels of B6 mice as early as at 4 weeks of age at stimulus frequencies of 8 kHz, 16 kHz, and 32 kHz. These features are basically in accordance with the previous report (Zheng et al., 2009). We also showed, for the first time, that decrease in DPOAE amplitudes in A/J mice occurred as early as 3 weeks of age, compared with the levels of B6 mice. DPOAE is produced directly by the sensory OHC, and abnormal DPOAE amplitudes at early stage imply early OHC impairment (Noben-Trauth & Johnson, 2009). Moreover, A/J mice suffer from progressive hair cell loss and degeneration of SGNs and stria vascularis after 2 weeks of age. Obviously, the pathologic impairment in the cochleae contributes to the progressive hearing loss in A/J mice.

We then tried to figure out the reasons for the pathologic impairment in A/J mice. Our results indicate that apoptosis in cochleae is related to early onset of hearing loss in A/J mice. Though the mechanisms leading to genetic hearing loss are not completely understood, caspase-family proteases function as important signals in the inner ear pathology. Apoptosis has been identified as the final common pathway in the degradation of the organ of Corti in several types of genetic hearing loss (Noben-Trauth et al., 2003; Op de Beeck et al., 2011; Laine et al., 2007; Niu et al., 2007; Noben-Trauth & Johnson, 2009; Moyer et al., 2011). Apoptosis is also involved in inherited deafness such as DFNB12 (Noben-Trauth & Johnson, 2009; Schwander et al., 2009; Han et al., 2012). Age-related loss of hair cells and SGNs through apoptosis has come from an association of aging with the expression of apoptosis-related proteins in the cochleae (Alam et al., 2001; Nevado et al., 2006). As A/J mice are a model of AHL, time course (from P1 to P56) caspase dependent and independent molecules were detected in this mouse. We show that mRNA levels of *caspase-3* and *caspase-9* in the inner ears of A/J mice were the highest at P1, indicating caspase-dependent apoptotic signals occur in A/J mice at early stages of inner ear development. The results of Z-VAD-FMK treatment improving hearing further support our idea that hearing loss in A/J mice is related to caspase-mediated apoptosis in the inner ears.

Second, our results suggest that decreased activity of *Cs* or decreased activity of *Cs* and CDH23 may lead to cell apoptosis in A/J mice. A/J mice and B6 share an *ahl* allele, but they show dramatic differences in the time courses of AHL. Mitochondrial dysfunction associated with excess reactive oxygen species (ROS) generation and decreased energy metabolism is thought to play a central role in many age-associated neurodegenerative diseases, including AHL (Jiang et al., 2007; Yamasoba et al., 2007; Someya et al., 2009; Someya & Prolla, 2010). *Cs* plays a decisive role in regulating energy generation and ROS production of mitochondrial respiration (Cheng et al., 2009). It was reported that A/J mice differ in *Cs* enzyme kinetics and catalytic properties from mice of other strains (Johnson et al., 2012). There is also a report that H55N caused low *Cs* activity (50% to 65% reduction) in skeletal muscle of A/J mice compared with other strains (Ratkevicius et al., 2010). Therefore, mutation in the *Cs* gene might be expected to have deleterious effects on mitochondrial function, leading to progressively impaired energy metabolism, continuous ROS production, oxidative damage, and eventual cell death (Balaban et al., 2005; Dirks et al., 2006). In fact, we did detect higher mRNA level of *catalase* at P1 in A/J mice (data not shown), indicating higher levels of ROS production, which may trigger mitochondrial-mediated apoptosis pathways at an earlier stage in the cochleae. Moreover, we show that transfection with *Cs* shRNA or cotransfection with *Cs* shRNA and *Cdh23* shRNA increases the levels of caspase-3 in HEI-OC1 cells. Though mutations in *Cdh23* and *Cs* lead to decreased activity of these proteins in A/J mice, which is different in principle from that of RNA interference, A/J mice may show phenotypes as the results of relative short of the active proteins. The results *in vitro* may, at least partially, aid in understanding that apoptosis may rise from decreased activity of *Cs* or decreased activity of *Cs* and CDH23 in A/J mice. However, knockdown of *Cdh23* alone fails to lead to an increase in caspase-3 *in vitro*, which is different from reports that mutations in *Cdh23* lead to apoptosis in the mouse cochleae (Schwander et al., 2009; Someya et al., 2009; Han et al., 2012). Several factors including the degrees of mRNA knockdown, the apoptosis pathways involved, and the possibility of compensative increase of antiapoptotic elements in the cells, and so forth should be taken into account.

Lastly, it should be mentioned that interaction of mutant *Cs* and *mt-Tr* may also be involved in the early onset of hearing loss. Genetic study showed that the mean ABR thresholds of B6.A-ahl4/ahl4 mice with A/J mtDNA were significantly higher than those with B6 mtDNA, but the average thresholds of B6 mice with A/J mtDNA were not statistically different from those of B6 mice with B6 mtDNA, indicating that hearing loss in *ahl4/ahl4* mice is exacerbated by the presence of A/J-derived mitochondria (Noben-Trauth & Johnson, 2009; Johnson et al., 2012). Therefore, interaction of *ahl4* with A/J-derived mitochondria is supposed to be involved in the hearing loss. However, there are still limitations in regulating mitochondrial gene transcription or expression *in vitro*.

## Summary

A/J mice showed earlier progressive hearing loss and more severe lesions in cochleae compared with B6 mice. Moreover, A/J mice displayed higher transcription levels of *caspases* in the inner ears. Further study revealed that knockdown of *Cs* alone or knockdown of *Cs* and *Cdh23* together upregulated caspase-3 in HEI-OC1cells. Finally, antiapoptotic treatment improved the hearing of A/J mice. The results suggest that apoptosis in cochleae contributes to the earlier onset of hearing loss in A/J mice.
